# Phenomic analysis of the honey bee pathogen-web and its dynamics on colony productivity, health and social immunity behaviors

**DOI:** 10.1371/journal.pone.0263273

**Published:** 2022-01-31

**Authors:** Renata S. Borba, Shelley E. Hoover, Robert W. Currie, Pierre Giovenazzo, M. Marta Guarna, Leonard J. Foster, Amro Zayed, Stephen F. Pernal

**Affiliations:** 1 Agriculture & Agri-Food Canada, Beaverlodge Research Farm, Beaverlodge, Alberta, Canada; 2 Department of Biochemistry and Molecular Biology, University of British Columbia, Vancouver, British Columbia, Canada; 3 Department of Biological Sciences, University of Lethbridge, Lethbridge, Alberta, Canada; 4 Department of Entomology, University of Manitoba, Winnipeg, Manitoba, Canada; 5 Département de Biologie, faculté des sciences et génie, Université Laval, Québec City, Québec, Canada; 6 Department of Biology, York University, Toronto, Ontario, Canada; King Khalid University, SAUDI ARABIA

## Abstract

Many pathogens and parasites have evolved to overwhelm and suppress their host’s immune system. Nevertheless, the interactive effects of these agents on colony productivity and wintering success have been relatively unexplored, particularly in large-scale phenomic studies. As a defense mechanism, honey bees have evolved remarkable social behaviors to defend against pathogen and parasite challenges, which reduce the impact of disease and improve colony health. To investigate the complex role of pathogens, parasites and social immunity behaviors in relation to colony productivity and outcomes, we extensively studied colonies at several locations across Canada for two years. In 2016 and 2017, colonies founded with 1-year-old queens of diverse genetic origin were evaluated, which represented a generalized subset of the Canadian bee population. During each experimental year (May through April), we collected phenotypic data and sampled colonies for pathogen analysis in a standardized manner. Measures included: colony size and productivity (colony weight, cluster size, honey production, and sealed brood population), social immunity traits (hygienic behavior, instantaneous mite population growth rate, and grooming behavior), as well as quantification of gut parasites (*Nosema* spp., and *Lotmaria passim*), viruses (DWV-A, DWV-B, BQCV and SBV) and external parasites (*Varroa destructor*). Our goal was to examine: 1) correlations between pathogens and colony phenotypes; 2) the dynamics of pathogens and parasites on colony phenotypes and productivity traits; and 3) the effects of social immunity behaviors on colony pathogen load. Our results show that colonies expressing high levels of some social immunity behaviors were associated with low levels of pathogens/parasites, including viruses, *Nosema* spp., and *V*. *destructor*. In addition, we determined that elevated viral and *Nosema* spp. levels were associated with low levels of colony productivity, and that five out of six pathogenic factors measured were negatively associated with colony size and weight in both fall and spring periods. Finally, this study also provides information about the incidence and abundance of pathogens, colony phenotypes, and further disentangles their inter-correlation, so as to better understand drivers of honey bee colony health and productivity.

## Introduction

Honey bees are an essential component of modern agriculture. In Canada alone, beekeepers produce approximately 40,000 tonnes of honey per year and provide $4–5.5 billion CDN in pollination services [1,2]. The worldwide economic value of pollination is estimated to be €153 billion [3], to which honey bees are the principal contributing species. Every year beekeepers struggle to remain profitable, a task made more difficult by the numerous pest/disease pressures on the Canadian honey bee population. During the winters of 2006–07 to 2020–21, Canadian beekeepers have lost an average of 26.0% of their colonies, representing an increase of at least 10% from historically acceptable levels [4]. One of the key factors affecting colony loss is the presence of multiple pathogens and parasites, as well as the background levels of their infective agents within hive substrates (*i*.*e*., wax, pollen, honey). Honey bee health is therefore challenged on many fronts, resulting in multifaceted and dynamic causes of colony mortality.

Several studies have shown the negative effect of viruses, bacteria, fungi, trypanosomes and mites on both individual bee and colony health [5–12]. For example, chronic Deformed Wing Virus (DWV) infections affect the longevity and behavior of individual bees, and ultimately lead to decreased colony fitness and performance [13]. Other infectious agents, such as *Nosema* spp. and sacbrood virus (SBV), cause changes in honey bee foraging behavior with *Nosema-*infected bees exhibiting precocious foraging or shifts in foraging preferences [14–17], and SBV-infected bees favoring nectar over pollen collection [14,15,18]. Although these effects may be indirect, they have great potential to negatively impact colony productivity and strength. The impacts of pathogen and parasite infections on colony productivity traits (e.g. honey production and brood population) and pre- and post-winter phenotypes (e.g. colony weight, cluster size) are still relatively undocumented, despite the expectation that they are predictive of colony outcomes.

The eusocial nature of honey bee colonies has afforded them great resilience. Though pathogens and parasites result in diseases and may affect bee behavior, honey bees have evolved remarkable capacity to counter these challenges at both the individual and colony level (*i*.*e*., individual vs. social immunity). These collective behavioral defenses, such as hygienic behavior, grooming behavior and propolis deposition [19–21], can reduce colony-level pathogen and parasite infections and improve colony health. Hygienic behavior is one of the most well-studied social behaviors in bees. Workers from hygienic colonies preferentially remove *Varroa*-infested pupae, as well as American foulbrood and chalkbrood-infected brood, more readily than workers from non-hygienic colonies [22–24]. Whether or not virus-infected brood is also effectively detected and removed by hygienic bees at the same rate as bacterial and fungal-infected brood, requires further evaluation [25,26]. Grooming is considered a principal immunity behavior because of its central role as a defensive behavior against parasitic mites. It is the main mite defensive behavior performed by *Apis cerana* [27], the original host of *Varroa destructor* [28]. During grooming, bees will physically remove mites from their own, or from a nest-mate’s body, often injuring mites and causing lethal damage to them. High intensity grooming coupled with a high proportion of damaged mites has been shown to be associated with lower mite infestation levels in bees [21,29]. Nevertheless, the relationship between grooming intensity (and/or damage) and levels of mite-transmitted viruses remains unknown.

An important and effective way to promote honey bee health and colony profitability is by improving local genetic stock though selecting for pathogen resistance and enhanced productivity. Beekeepers often use a combination of field-based assays to determine colony phenotypes and select breeder colonies. Additionally, new genomic and proteomic selection tools that are becoming available to beekeepers have the potential to encourage larger and more sophisticated breeding programs [30]. As part of a large-scale study across Canada to discover genetic breeding markers, we evaluated 12 economically-valuable and heritable traits (colony phenotypes) to determine their associations with several important pathogens, so as to better understand drivers of colony health and productivity. Using the comprehensive data collected, we examined the following relationships: 1) the correlations between pathogens and colony phenotypes; 2) the dynamics of pathogens and parasites on colony phenotypes and productivity traits; and 3) the effects of social immunity behaviors on colony pathogen load. Understanding these relationships will enable local queen producers to make informed management decisions when selecting and breeding colonies to be healthy and productive, and otherwise improve commercial management decisions made by beekeepers.

## Materials and methods

**Fig 1:**
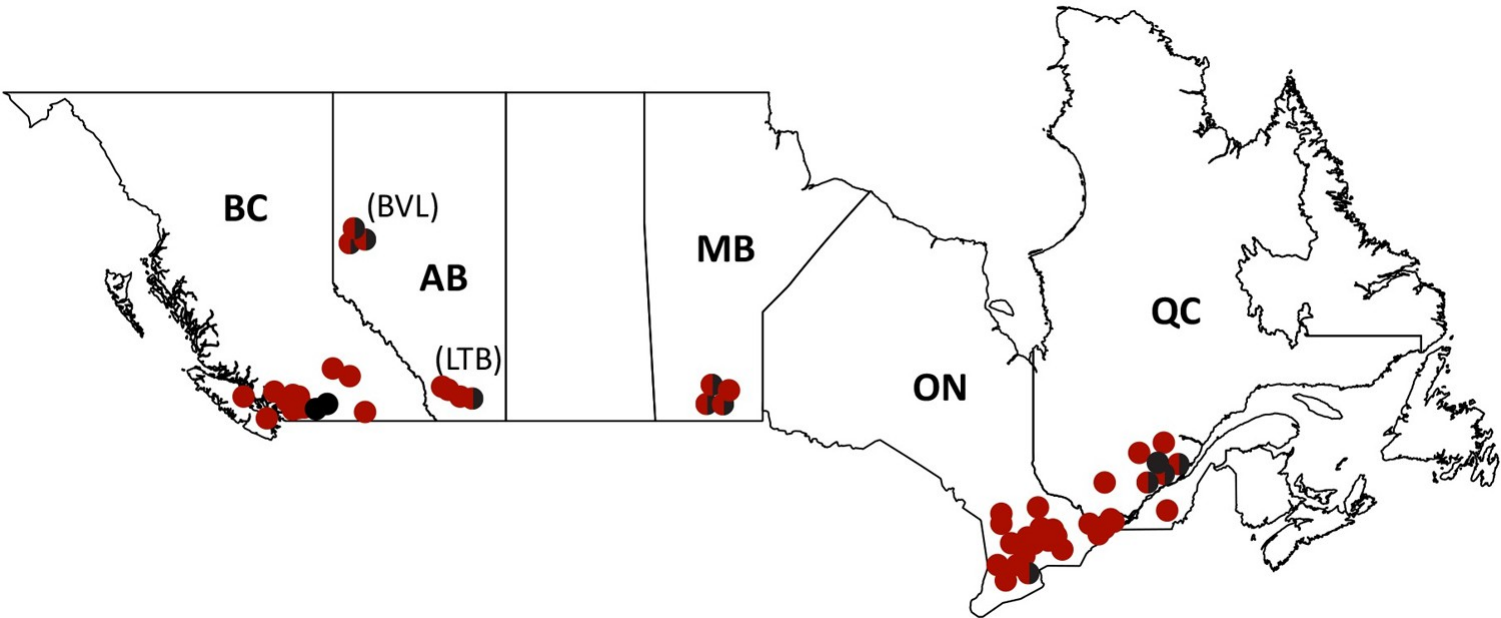
**Map of Canadian provinces (territories not shown) illustrating the geographical distribution of the apiaries in the experimental year of 2016–2017 (red circles) and 2017–2018 (black circles).** Red and black circles represent yards used in both experimental years. Participating provinces are identified with their two-letter abbreviations (BC = British Columbia, AB = Alberta, MB = Manitoba, ON = Ontario, QC = Quebec). The two distinct locations sampled in the province of Alberta are identified by three-letter abbreviations (BVL = Beaverlodge, LTB = Lethbridge).

This experiment was conducted across five provinces in Canada (Table 1; Fig 1) from May 2016 through April 2017, and was repeated from May 2017 to April 2018. A total of 1,025 honey bee colonies were established in May 2016 as “package” style units or from beekeeper-owned colonies (experimental year 1; see details below). In May 2017, a second set of 496 colonies were established as packages to serve as a replicate of this experiment (experimental year 2) for a total of 1,521 colonies across both years. In both years, 1-year-old queens of diverse genetic origin, predominantly obtained from local Canadian queen breeders, were used, thereby providing a large representative subsample of the Canadian commercial honey bee population. Participants did not sample large numbers of colonies headed by imported queens from an individual breeder.

**Table 1:** Total number of Intensive Management (IM) and Standard Management (SM) colonies, followed by the number of apiary sites (in brackets), within each province and experimental season.

	2016–2017		2017–2018
Province	IM	SM	IM
British Columbia	0	204 (12)	79 (2)
Alberta-Beaverlodge	147 (3)	0	100 (3)
Alberta-Lethbridge	51 (2)	51 (2)	59 (1)
Manitoba	192 (4)	0	120 (3)
Ontario	0	200 (22)	47 (1)
Quebec	24 (1)	147 (6)	91 (4)
**TOTALS**	**414 (10)**	**602 (42)**	**496 (14)**

### General colony management and establishment

In experimental year 1, a two-tier colony management approach was incorporated to allow us to collect more precise phenotypic data from a large subset of the colonies:

#### Intensive Management (IM)

Colonies in this tier were established by shaking 1.0 kg of adult bees into single, full-depth Langstroth boxes with nine empty frames and the original, 1-year-old, queen. These colonies were managed by research teams, which had full control over disease treatment and colony management. Colonies from the IM tier were managed as single brood chamber units and were not treated for *Varroa* mites or *Nosema* spp. until the fall. To prevent swarming, colonies were provided with sufficient honey supers (Langstroth deeps) at all times and had all swarm cells removed approximately every second week. Supplemental feeding was provided in the spring (1:1 v/v sucrose syrup and 0.5 kg pollen patties containing 15% pollen; Global Patties Canada, Airdrie, AB, Canada) and fall (1:1 v/v sucrose syrup), as would be typical of management in a commercial beekeeping operation. After the last fall assessment, all colonies were wintered indoors in temperature-controlled rooms at 4–5°C [31].

#### Standard Management (SM)

Colonies in this tier were owned by collaborating beekeepers. All SM colonies had the original (overwintered) 1-year-old queen or a newly-bred queen that would have been laying for at least 50 days prior to the first data collection. Colony size was standardized at day 0 of the experiment within each apiary, but varied among and within beekeeping operations. These colonies were managed by cooperating beekeepers, consequently, intensive manipulation was not always permitted. Colonies from the SM tier were managed as either single or double brood chamber colonies. Disease an mite treatment, as well as spring and fall supplemental feeding, were provided at the cooperating beekeeper’s discretion. Beekeepers were allowed to remove frames of brood/bees from colonies as part of their normal management practice to prevent swarming. Wintering management (indoors or outdoors, insulation, timing) also varied among beekeepers.

For SM colonies, manipulations and management (e.g., disease treatment, overwintering method) were always standardized within apiary sites, irrespective of differences in management practices among and within beekeeping operations. In the event of swarming or queen loss, colonies were removed from the experiment.

In the second experimental year, all colonies were part of the IM tier and therefore followed similar colony establishment, management and phenotyping protocols as the IM tier colonies in the first experimental year. Among the two years, the IM-managed apiaries differed only with respect to wintering techniques. IM colonies in British Columbia and Ontario in the second year were overwintered outdoors while all other IM colonies were overwintered indoors in both years, reflecting local practices.

### Initial *Varroa* population level standardization

One of the main differences between the two management tiers was the initial *Varroa destructor* population at time of colony establishment. Colonies in the IM tier were intensively manipulated to contain a standardized initial *V*. *destructor* phoretic infestation rate, varying between 0.5% - 2% per 100 adult bees, as a way to normalize the effect of mite population on the other variables. Colonies with low *Varroa* mite levels (infestation lower than 0.5%) were inoculated with mites from highly infested colonies using a CO_2_ “shake” [32] or icing sugar dusting method [33]. Mites found on the paper-lined cover tray were collected with the tip of a soft paint brush, placed in Petri dishes lined with moist wipes and introduced into the colonies by placing the moist wipe containing mites on the top bar of brood chamber frames.

### Honey bee sampling and trait assessment

In both years, colonies were evaluated after establishment in May/June until April of the following year. Samples collected in the field were stored in coolers with ice or dry ice (depending on the sample storage temperature required). Upon arrival at the laboratory, samples were immediately stored at -20°C or -80°C (samples for virus analysis and downstream molecular biology) until analyzed (Table 2).

**Table 2:** List of phenotypic variables measured, time of sample collection, targeted sample size per colony, method of assessment and management tier sampled.

Phenotypic Variables		Unit	Time of collection	# Bees sampled	Assessment method	Management tier
**Pathogen load**	***Nosema* spp.**	Spores/bee	August	60	Microscopy	IM and SM
	***Lotmaria passim***	Copy number/bee	August	60	qPCR	IM and SM
	**Viruses (BQCV, DWV-A, DWV-B, SBV)**		October and April of the following year	60	qPCR	IM and SM
	***Varroa destructor* infestation level**		May	500	Alcohol wash	IM and SM
		Varroa/100 bees	June, August, October and April of the following year	300		
			September–October	-	Sticky boards	IM
**Social immunity / Parasite resistance**	**Hygienic behavior**	%	May/June September		Freeze-kill assay	SM IM
	***Varroa destructor* resistance behavior**		May–August		Instantaneous Mite Population Growth Rate	IM
	**Grooming behavior**		August		Mite damage and mite fall	IM and SM
**Productivity traits**	**Total honey production**	Kg	June–September	-	Total weight of honey produced	IM
	**Instantaneous honey production**	Kg	July	**-**	Gross colony weight gain	IM and SM
	**Mid-summer sealed brood population**	Cell	August	**-**	Photographic assessment	IM
**Pre- and post- winter phenotypes**	**Colony weight and cluster size**	Kg / interframe spaces	October and April of the following year	-	Total weight and number of filled interframe spaces	IM and SM

Phenotypic variables were assessed as follows:

#### *Varroa destructor* resistance

Following previously published methods [34], *Varroa destructor* resistance was assessed using the Instantaneous Mite Population Growth Rate (IMPGR) calculation, estimated as follow: P_2_ = P_1_ x *e*^*rn*^; where *r* is the growth rate per week after *n* number of weeks (*i*.*e*., 13–17 weeks) and *e* is the base of the natural logarithm. Initial mite population (P_1_) was calculated at the time of colony establishment using a combination of alcohol wash and manual mite inoculation data. A slightly modified alcohol wash method from De Jong et al. [35] was used, in which each alcohol container with bees was shaken at 130 RPM for 5 min using an orbital shaker. Successive rounds of agitation were repeated until no additional mites could be recovered. The final mite population (P_2_) was assessed for 6 weeks following a clean-up treatment using amitraz strips (Apivar^®^; Veto-pharma, Palaiseau, France). To collect dead mites after miticide treatment, an adhesive-coated board (Varroa Mite Sticky Board, Bee Maid, Spruce Grove, AB, Canada) was inserted beneath each screened bottom board. Currently, there is no evidence of amitraz resistance in Canada, and to our knowledge, the population used in this study had no resistance to amitraz.

#### *Varroa destructor* phoretic load

*V*. *destructor* levels were measured using the alcohol wash method [35] four times during each experimental year: at colony establishment (May), before and after miticide treatment application (late August and October, respectively), and after winter (April of the following year). With the exception of the initial *V*. *destructor* sampling in May of both experimental years, where a sample of 500 adult worker bees was taken, samples of 300 bees were collected thereafter from brood frames to assess mite infection level. The number of mites per 100 adult workers was recorded for each sampling period and calculated as a percentage of infestation. For colonies in the IM tier, sticky boards were used on the bottom boards of colonies to assess final mite population (as described previously).

#### Honey production

Two methods were used to assess the amount of honey produced by a colony. To estimate the total honey production, each honey super weight was recorded using a battery-operated digital platform (*e*.*g*., model GBK 260A, Adam Equipment, Oxford, CT, USA) before placing it on a colony and net honey production per box was calculated at the time of honey extraction. As an additional measurement of colony honey production, total gross weight of the entire colony and all of its components (without any rocks, bricks, etc. on the lid) was recorded in the field using a battery-operated digital platform or hanging scale (Optima Digital Crane Scale, Model WGB1483661, Global Industrial Canada, Toronto, ON, Canada) at the beginning and end of a two-week period during peak honey flow [36], referred here as ‘instantaneous’ honey production.

#### Hygienic behavior (HB)

Colonies were evaluated using the liquid nitrogen freeze-kill brood assay [37] as per methods described in Guarna et al. [38], with the assay repeated 3–10 days after the first assessment. HB was measured as the proportion of pupae completely removed after 24 hr of freezing. Colonies were considered hygienic when the proportion of freeze-killed brood removed by workers during round 1 and round 2 of this assay averaged 0.95 or higher in a 24 hr period. HB was assessed before the honey flow in SM tier colonies (*i*.*e*., April) and after the honey flow in IM tier colonies (*i*.*e*., late August and early September).

#### Grooming behavior (GB)

*V*. *destructor* natural mite fall was collected on plastic-coated freezer paper (Reynolds Kitchens®, Richmond, VA, USA) covered with petroleum jelly placed on a hardboard panel and introduced beneath the screened bottom board of the colonies for two consecutive 72-hour periods in the fall (before miticide treatment). In the lab, all mites were carefully removed from petroleum jelly-coated boards and stored in 70% ethanol until analysis. Colonies with a sample size greater than 200 mites had a subsample of 200 mites randomly selected for analysis. Mites were assessed for signs of damage under 32-50x magnification (Leica MS5, Leica Microsystems, Wetzlar, Germany). Both the dorsal and ventral sides of the mites were examined for signs of damage to their mouthparts, legs, and ventral shields (dented idiosoma were not considered a sign of damage). The proportion of damaged mites was calculated by combining the total number of mites with any signs of damage and dividing that by the total number of mites analyzed (“grooming mite damage”). We also calculated the proportion of natural mite fall per day standardized to the total mite population by dividing the total number of mites collected on the petroleum jelly-coated boards by the final mite population (P_2_) and then dividing it by three (*i*.*e*., number of days; “grooming mite drop”).

#### Mid-summer sealed brood population

Using a high resolution camera (D7200 Nikon, Tokyo, Japan) equipped with micro VR lens (Nikkor 105 mm, Nikon, Tokyo, Japan) and filter (Promaster 62 mm 1A Skyfilter), an image of each side of a frame containing worker sealed brood was captured and later analysed using Honeybee Complete software (version 4.2, WSC Scientific GmbH, Heidelberg, Germany). Sealed worker brood cells were electronically recognized by the software, with manual corrections made by a technician who performed quality performance checks on each image. The total number of sealed brood cells were recorded for each colony. Sealed worker brood population was measured once between late July and early August, when populations were at peak levels.

#### Pathogen sampling

*Nosema* spp., *Lotmaria passim* and honey bee RNA viruses sampling were conducted in the fall (late August), prior to colony overwinter preparation, as colony health in early fall is critical for overwintering success [39]. Virus sampling was repeated in the following spring from surviving colonies. The five honey bee viruses quantified in our study were selected based on their strong association to colony mortality and overwinter success [9,12].

*Nosema spp*.. Samples of 60 adult worker bees collected from a honey frame in the periphery of the brood nest were homogenized in 60 mL of 70% ethanol (1 mL/bee) and an aliquot of 6 μL was analyzed microscopically twice for the presence of *Nosema* spp. spores [40].

*Lotmaria passim*. From the same pool of homogenized samples used to assess *Nosema* spp. infection level, a 200 μL aliquot was collected, centrifuged and pelleted to quantify *L*. *passim* load by real-time PCR. Following centrifugation, ethanol was aspirated from the pellet and left to dry at room temperature to evaporate any remaining ethanol. Genomic DNA was extracted using NucleoSpin®Tissue kit following manufacturer instructions (Macherey-Nagel Gmbh & Co. KG, Düren, Germany). Quantification of *L*. *passim* load was carried out using previously published primers (S1 Table) and SSoAdvanced™ Universal SYBR® Green Supermix (Bio-Rad Laboratories, Hercules, USA). Amplification assays were performed in a CFX384 Touch™ Real-Time Detection System (Bio-Rad Laboratories, Hercules, USA) by triplicate employing 60 ng of gDNA. Standard curves were prepared from plasmids harboring the target/reference gene amplicons with copy numbers diluted from 10^7^ to 10^2^. PCR conditions were 3 min at 98°C for initial denaturation/enzyme activation followed by 40 cycles of 10 seconds at 98°C and 20 seconds at 60°C. Specificity was checked by performing a melt-curve analysis from 65–95°C with increments of 0.5°C at 2 seconds/step.

*Viruses*. Samples of 60 honey bees collected from the outer frames of the brood chamber were homogenized in 12 mL of GITC buffer [41]. An aliquot of 200 μL was used to isolate total RNA using NucleoSpin®RNA kit following manufacturer instructions (Macherey-Nagel Gmbh & Co. KG, Düren, Germany). cDNA was synthesized from 800 ng of total RNA for 20 minutes at 46°C in a final volume of 20 μL using the iScript cDNA synthesis kit (Bio-Rad laboratories, Hercules, USA). cDNA was diluted with 60 μL of molecular grade water to a total of 80 μL, from which three μL were used for qPCR quantification. Quantification of Black Queen Cell Virus (BQCV), Deformed Wing Virus type A (DWV-A), Deformed Wing Virus type B (DWV-B), Sacbrood Virus (SBV), and Israeli Acute Paralysis Virus (IAPV) were determined by real-time PCR using previously published primers (S1 Table) and SSoAdvanced™ Universal SYBR® Green Supermix (Bio-Rad Laboratories, Hercules, USA). Amplification assays were performed in triplicate employing approximately 30ng of cDNA in a CFX384 Touch™ Real-Time Detection System (Bio-Rad Laboratories, Hercules, USA). Standard curves were prepared from plasmids harboring the target/reference gene amplicons with copy numbers diluted from 10^7^ to 10^2^. PCR conditions were 3 min at 95°C for initial denaturation/enzyme activation followed by 40 cycles of 10 seconds at 95°C and 30 seconds at 60°C (except IAPV, where annealing/extension was 45 seconds at 60°C). Specificity was checked by performing a melt-curve analysis from 65–95°C with increments of 0.5°C at 2 seconds/step. Quantitative PCR data for the five viruses and *L*. *passim* were analyzed with the CFX Manager™ Software (Bio-Rad Laboratories, Hercules, USA) and exported to an Excel spreadsheet (Microsoft Corporation, Redmond, USA) to calculate copy numbers.

#### Overwintering success

Gross colony weight and colony cluster size were recorded at two time points: in the fall, immediately before colonies were insulated for wintering outdoors, or prior to being moved indoors into a wintering facility; and in the spring, immediately after insulated wraps were removed from outdoor wintered colonies or after colonies were moved out of wintering rooms. Total gross colony weight was recorded using a battery-operated digital platform scale (e.g. Adam Platform-weighing Scale, 150 kg capacity, or equivalent). The cluster score was evaluated early in the morning or when the temperature was < 5°C, to ensure the cluster had not broken and flight had not yet begun. Colonies with any number of surviving bees plus a queen were recorded as surviving in April of the following year.

### Statistical analysis

All response variables (Table 2) were tested for normality and homogeneity of variance. Final mite population, instantaneous honey production, fall and spring colony weight, *Nosema* spp. spore count, *L*. *passim* and viruses copy numbers were log_10_ transformed. *V*. *destructor* phoretic load (mites per 100 bees) was transformed into proportion data dividing by 100. Total honey, as well as fall and spring colony cluster size were square root transformed, and mid-summer sealed brood population was divided by 10 000. To allow for comparison between both experimental years, data for pre- and post-winter phenotypes from colonies managed as double brood chamber units in the first year were excluded from the analysis. All data analyses were performed using R software [42].

Simple correlation analyses of pathogens and colony-level phenotypes was performed using Spearman’s Rho statistic with Bonferroni correction for multiple tests, using the *corrplot* package and *cor* function.

Response variables were compared among locations using ANOVA with apiary as a random variable, followed by Tukey’s HSD test. For models including pre- and post-winter phenotypes, colony size (*i*.*e*., single *vs*. double brood chamber) was included as a fixed factor and found to have a significant effect.

Linear regression analysis was used to investigate the effect of pathogen load on productivity (*i*.*e*., sealed brood and honey production) and colony-level pre- and post-winter phenotypes (*i*.*e*., fall and spring colony weight and cluster size). Pathogen abundance (explanatory variable) was regressed separately against each phenotype (dependent variable) with location added as a random factor and apiary nested within location. Where appropriate, colony size (*i*.*e*., single or double brood chamber) and tier were added as fixed factors. We also investigated the effect of social immunity/parasite resistance (*i*.*e*., hygienic behavior, correlates of grooming and *V*. *destructor* resistance) on pathogen load. In this case, social immunity/parasite resistance were included in the model as explanatory variables and fitted separately to each pathogen. Similarly, location was added as a random factor with apiary nested within location, and colony size and tier were added as fixed factors where appropriate. All linear regression analyses were carried out using *lm4* package.

## Results

### Colony-level phenotypes and pathogen abundance levels

**Fig 2:**
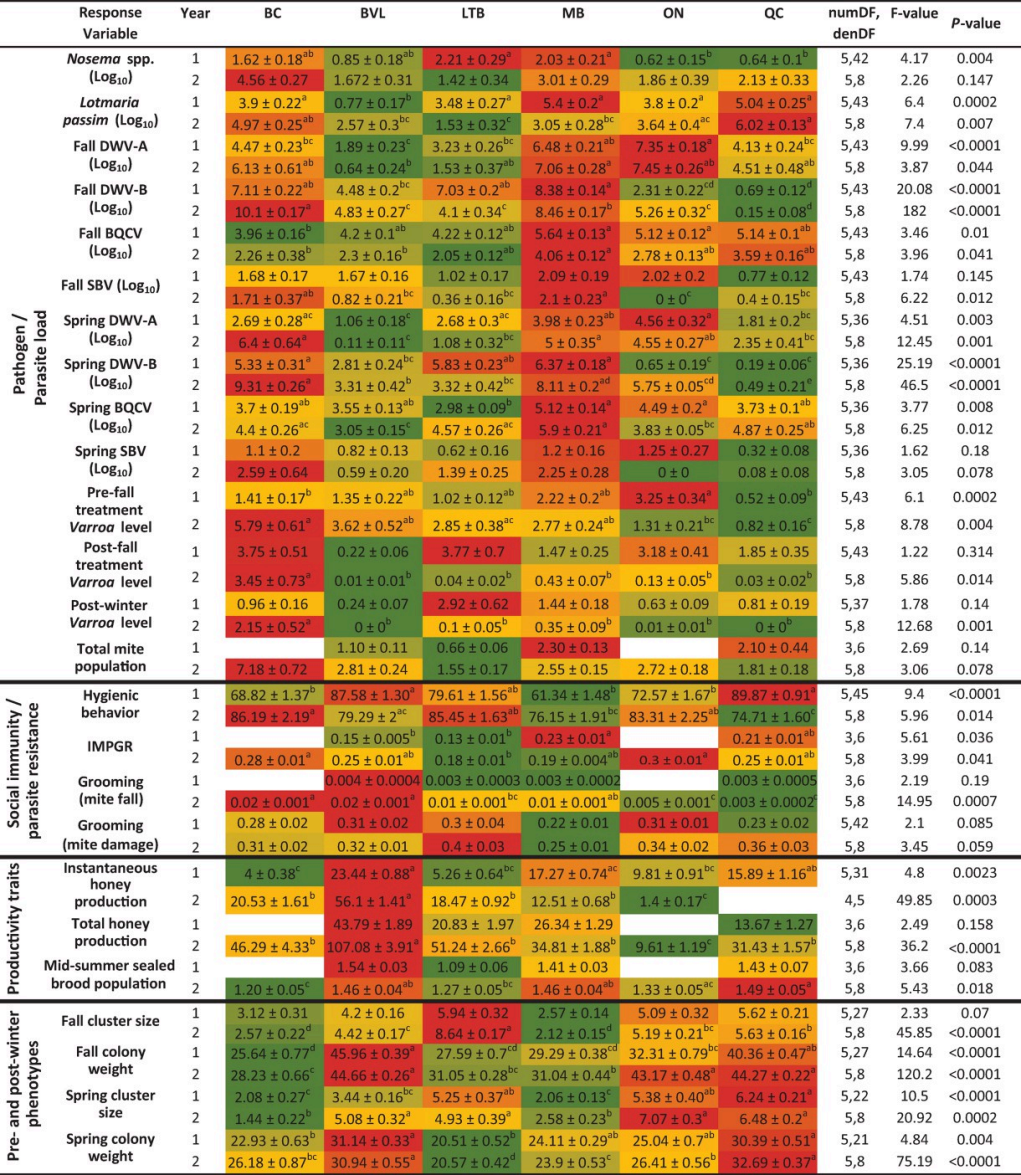
Pathogen/parasite load and phenotypic assessment value averages (± SEM) for each location during the experimental years of 2016–2017 (year 1) and 2017–2018 (year 2). Pathogen load averages for viruses, *Nosema* spp., and *Lotmaria passim* are reported on the same scale the data was analysed (log_10_). Total mite population data was divided by 1 000 and mid-summer sealed brood population by 10 000. All other variables are on their original scale. Statistically significant differences of response variables among locations are denoted with different letters. A color gradient was used to represent relative mean changes within each experimental year (not statistical differences), for each variable, from green (lowest) to red (highest). Measurement units are as follows: *Nosema*, spores/bee; *Lotmaria passim* and viruses, genome copies/bee; *Varroa* level, mites/100 bees; total mite population, total count; hygienic behavior, percentage of cells removed after 24 hrs; instantaneous mite population growth rate (IMPGR), mite growth rate per week; grooming (mite drop), proportion of mite fall per day; grooming (mite damage), proportion of damaged mites; colony weight, instantaneous and total honey production, weight (kg); mid-summer sealed brood population, total number of worker sealed brood cells; cluster size, total full frame spaces. Blank cells in year 1 (*e*.*g*., BC total honey production) are a result of unmeasured phenotypes in the standard management group.

In each location, an effort was made to sample, to the extent possible, bee genetics derived from local queen breeders. To determine if there was a spatial and temporal distribution pattern of pathogen abundance and spatial pattern of colony phenotypic expression, we compared the means of response variables (± SEM) from each location within each experimental year (Fig 2).

#### Pathogen load

The abundance of eight pathogens was measured each year, including one assessment of *Nosema* spp. and *L*. *passim*, two assessments (spring and fall) of five virus levels (DWV-A, DWV-B, BQCV, SBV and IAPV), and four assessments of *V*. *destructor* phoretic levels (pre- and post- fall miticide treatment, post-winter and total mite levels; Fig 2). IAPV was only detected by quantitative PCR in a very small portion of the samples (3%, 1% and 2% in the fall of 2016, spring and fall of 2017, respectively); therefore, IAPV abundance data was not included in the analyses. *Nosema* spp. and *L*. *passim* levels were assessed once during each experimental year, in the fall, prior to any disease treatment being applied to the colony. Although the number of *Nosema* spores per bee were significantly different among locations in the first year, *Nosema* levels were considered low in both years as average infection levels were below the nominal threshold of 1 million spores per bee at all sites [43]. The trypanosome *L*. *passim* was found in all locations, in both years, with lower levels in Beaverlodge in year 1.

Levels of DWV-A were significantly lower in Beaverlodge compared with Manitoba and Ontario in the fall and spring of year 1, lower than Manitoba in the fall of year 2, and lower than British Columbia, Manitoba and Ontario in the spring of year 2. For DWV-B, colonies in Quebec and Ontario had the lowest levels in the fall of year 1, and in the spring of the same year, Quebec and Ontario had levels similar to Beaverlodge but lower than British Columbia, Lethbridge and Manitoba. In year 2, Quebec had the lowest DWV-B levels of all locations in both fall and spring, whereas highest levels were in British Columbia. Levels of BQCV were higher in Manitoba than British Columbia in the fall year 1, and in the spring of the same year, levels for Manitoba were greater than Lethbridge. In year 2, BQCV levels in the fall were greater in Manitoba than British Columbia and Beaverlodge, while in the spring levels for Manitoba were greater than Beaverlodge and Ontario. Overall, the majority of pathogen loads (total *Nosema* spp. count and *L*. *passim*, DWV-A, DWV-B, BQCV and SBV abundances) were greatest in colonies from Manitoba in both experimental years, for both fall and spring time periods (Fig 2).

Viral levels were assessed in the fall (before colonies were insulated or moved indoors for the winter) and in the spring (from surviving colonies), and compared between the two seasons so as to understand viral seasonal dynamics. In the first year, all four viral levels significantly decreased from fall to the following spring (Table 3). In the second year, similar seasonal dynamics were observed for DWV-A and DWV-B levels. In contrast, BQCV levels were significantly higher in the spring of 2018 compared with its levels in the fall of 2017 and SBV levels were similar in the fall and spring.

**Table 3:** Viral seasonal variance during experimental year 1 and 2.

	2016–2017					2017–2018				
	Fall	Spring	DF	*F*-value	*p*-value	Fall	Spring	DF	*F*-value	*p*-value
	(copy number)					(copy number)				
DWV-A	4.49 ± 0.13	2.79 ± 0.11	1,120	184.3	<0.0001	4.39 ± 0.23	3.05 ± 0.19	508	42.0	<0.0001
DWV-B	4.71 ± 0.14	3.47 ± 0.13	1,120	168.0	<0.0001	5.14 ± 0.22	4.74 ± 0.22	508	5.98	0.014
BQCV	4.72 ± 0.06	4.02 ± 0.07	1,120	100.5	<0.0001	3.02 ± 0.09	4.55 ± 0.11	508	176.3	<0.0001
SBV	1.36 ± 0.07	0.87 ± 0.07	1,120	30.2	<0.0001	0.94 ± 0.11	1.07 ± 0.12	508	0.89	0.35

Significant differences between fall and spring abundances were compared by ANOVA. Average of overall abundance levels are reported for each virus by season, along with the degrees of freedom, *F*‐value and *p*‐value.

*Varroa* mite phoretic loads were assessed before and after miticide treatment in the fall, and again in the spring, following winter. We found that, in both years, colonies in British Columbia had levels above the fall recommended economic threshold of 3% subsequent to miticide treatment [44]; this was also true for colonies in Lethbridge and Ontario, only during year 1. Post-winter, colonies from Lethbridge and Manitoba showed phoretic mite levels above the spring economic threshold (1%) in the first experimental year and British Columbia in the second year (Fig 2).

#### Social immunity/parasite resistance

Hygienic behavior was measured as the percentage of frozen pupae completely removed after 24 hours. In year 1, Quebec and Beaverlodge had significantly greater mean hygienic removal compared to Manitoba, British Columbia and Ontario, with Lethbridge being intermediate (Fig 2). In contrast, Quebec had significantly lower mean hygienic removal in the second year compared with British Columbia, Lethbridge and Ontario, thereby showing no clear spatial pattern of expression. Instantaneous mite population growth rate (IMPGR) was used to assess a colony resistance behavior to *V*. *destructor*. Colonies in Lethbridge showed the lowest IMPGR (*i*.*e*. the most resistant) in both years of the experiment, and were significantly lower than Manitoba in year 1, and British Columbia and Ontario in year 2. Grooming behavior was assessed by calculating the proportion of mite fall per day standardized to the total mite population during a 3-day observation period, as well as the proportion of mites showing signs of damage to their mouthparts, legs, and ventral shields. Our data show a significant difference in mite fall among locations during the second experimental year only. Colonies from Quebec and Ontario had the lowest proportion of mite fall per total mite population (*i*.*e*. poorer groomers) compared with colonies in British Columbia, Beaverlodge and Manitoba. No apparent difference in proportions of damaged mites was detected among locations.

#### Productivity traits

Two measurements of honey production (instantaneous and total) were taken from all IM colonies in both years, whereas total honey production was only measured from SM colonies in year 2. Colonies from Beaverlodge had greater instantaneous honey production than those in British Columbia, Lethbridge or Ontario in year 1, and exceeded all locations in year 2 (Fig 2). For total honey production, Beaverlodge far exceeded all locations in year 2, with a similar trend but no statistical separation in year 1. Also in year 2, colonies from Beaverlodge, Manitoba and Quebec had significantly greater mid-summer sealed brood populations compared to colonies in British Columbia.

#### Pre- and post- winter phenotypes

Colony weight and cluster size were measured in the fall (before colonies were insulated or moved indoors for wintering) and in the spring from surviving colonies. Though no differences were seen in year 1, in year 2, colonies in British Columbia and Manitoba had the smallest cluster sizes in the fall, while Lethbridge had the largest (Fig 2). Our results also show that in both years, colonies from British Columbia, Lethbridge and Manitoba had fall colony weights below the recommended gross weight for overwintering success [31] and were statistically inferior to the gross colony weights from Beaverlodge and Quebec. In the spring of each following year, the smallest cluster sizes were seen from British Columbia and Manitoba, while colony weights were greatest in Beaverlodge and Quebec. However, it is important to point out that regional differences in regards to average winter temperatures and winter duration will influence the amount of supplemental feed, and therefore, fall colony weight, required prior to overwintering colonies.

### Pairwise correlation of pathogen abundances and colony phenotypes

**Fig 3:**
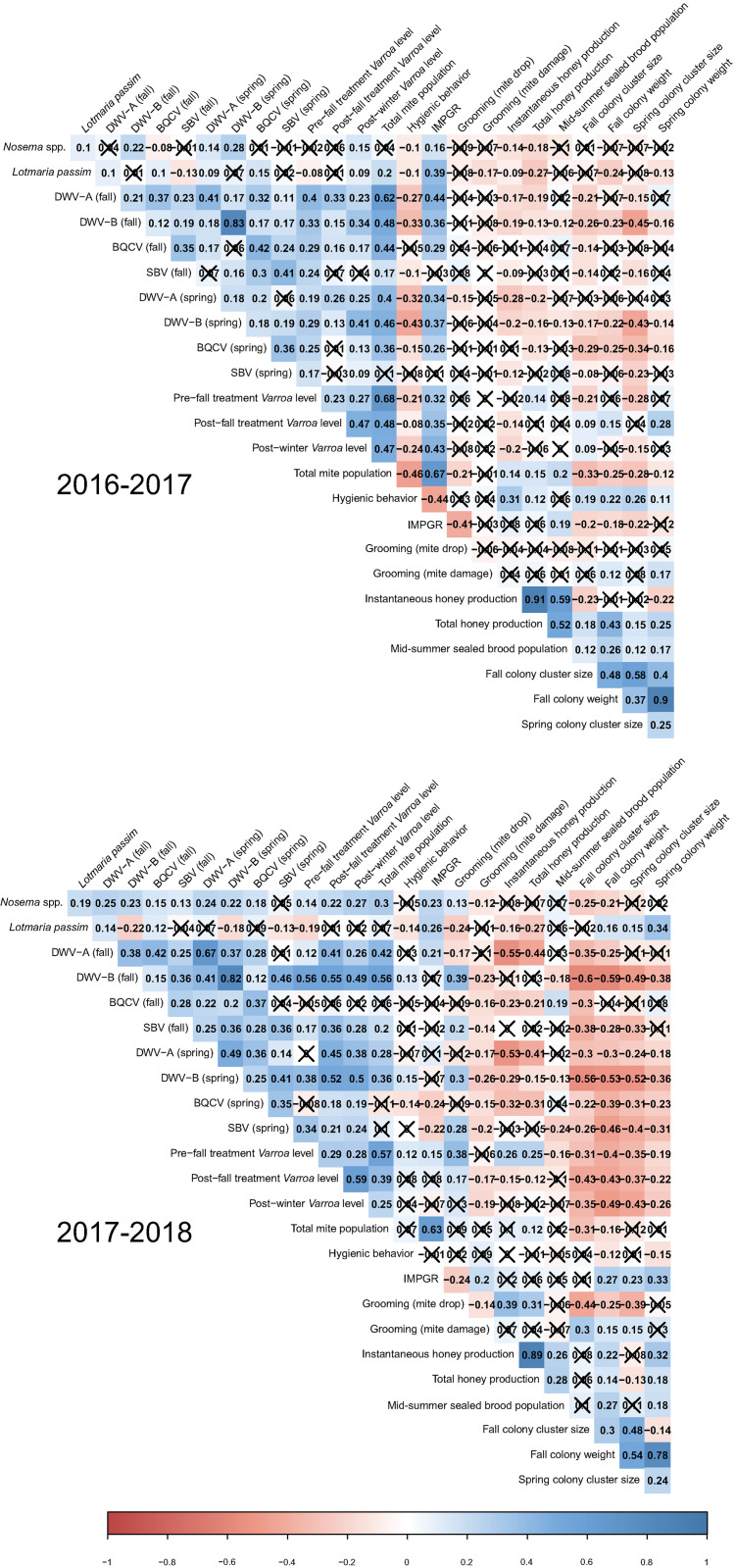
Pathogen and colony phenotypes correlation matrix using Spearman’s rho statistic with Bonferroni correction for multiple tests. Correlation coefficients (reported as R values) are shown for each pair-wise comparison. Statistically insignificant estimates (*P* > 0.05) are marked with an ‘X’. Shaded blue cells represent positive correlations and red cells represent negative correlations. Darker hues indicate stronger correlations as indicated by the correlation color gradient.

Honey bees act as a host for a multitude of pathogens and parasites, each with its own seasonal population dynamics, that have the potential to negatively impact colony health and productivity. To explore the dynamics of these pathogens, the interrelationship of colony phenotypes, and the potential effects that pathogen abundances have on colony phenotypes, we calculated the simple correlations between each pair of variables (Fig 3). Out of the 91 pathogen pairs, 72 were significantly correlated in the first year and 74 were significantly correlated in the second year. The strongest positive correlations within each study year were between fall and spring abundance levels of DWV-B (R = 0.83, *P*<0.001 (year 1); R = 0.82, *P*<0.001 (year 2)). In contrast, fall levels of SBV and *L*. *passim*, as well as fall levels of DWV-B and *L*. *passim* showed the strongest negative correlations in the first and second year, respectively (R = -0.13, *P*<0.001 (year1); R = -0.22, *P* <0.001 (year2)). Over both experimental years, 56 pathogen abundance pairs showed consistently positive and significant correlations, and only one pair, *L*. *passim* and pre-fall treatment *Varroa* levels, was consistently negatively correlated, indicating little or no competition among most of these pathogens.

We also examined the relationship between 55 pairs of colony-level phenotypes (Fig 3). We found 33 and 32 pairs to be significantly correlated in the first and second year, respectively. As we had anticipated, the strongest positive correlation pair in both years was between instantaneous and total honey production (R = 0.914 and R = 0.89, *P* <0.001). Overall, the strongest negative correlations were observed between hygienic behavior and IMPGR in the first year (R = -0.44, *P* <0.001), and between fall cluster size and grooming behavior (mite drop) in the second year (R = -0.44, *P* <0.001). Fourteen out of 55 colony phenotype pairs showed statistically significant correlations in both experimental years. Out of these 14 pairs, only one was negatively correlated, IMPGR and grooming behavior (mite fall), suggesting that grooming may play an important role in the colony resistance to *Varroa*.

Correlations between pathogen abundance and colony phenotypes help untangle the relationship between these variables. We identified 82 (year 1) and 98 (year 2) significant correlations out of 154 pairs of pathogen abundance and colony phenotypes. We found that IMPGR was highly correlated with total mite population in both years (R = 0.67 and R = 0.63, *P* <0.001). However, this relationship was expected because total mite population data is used in the calculation of a colony’s IMPGR. Furthermore, we found that fall levels of DWV-B had the highest negative correlation with spring cluster size in year 1 (R = -0.45, *P* <0.001), and fall cluster size in year 2 (R = -0.6, *P* <0.001). A comparison of the results between years shows that 44 pairs have significant and similar correlation directions in both years. From these 44 pairs, seven were between social immunity/parasite resistance behaviors, where all five correlations between pathogen abundances and increased IMPGR (*i*.*e*., lower *Varroa* resistance) were positive and the two correlations between pathogen abundance and hygienic behavior were negative. All but two of the 14 significant correlations between pathogen abundances and productivity traits were negative. Counterintuitively, the only two positive correlations were between total honey production and *V*. *destructor* variables. The remaining 23 significant correlation pairs were all negative correlations between pathogen abundances and pre- and post-winter phenotypes.

### Linear regression models of pathogen abundances and colony phenotypes

To further examine the relationship between pathogen abundance (*i*.*e*., *Nosema* spp., *L*. *passim*, DWV-A, DWV-B, BQCV, SBV, *V*. *destructor* loads) and colony-level phenotypes (*i*.*e*., social immunity/parasite resistance behaviors, colony productivity traits, pre- and post-winter phenotypes), we used linear regression models to explain colony phenotype patterns based on pathogen abundance, and to test if social immunity behaviors could significantly predict pathogen abundances.

**Fig 4:**
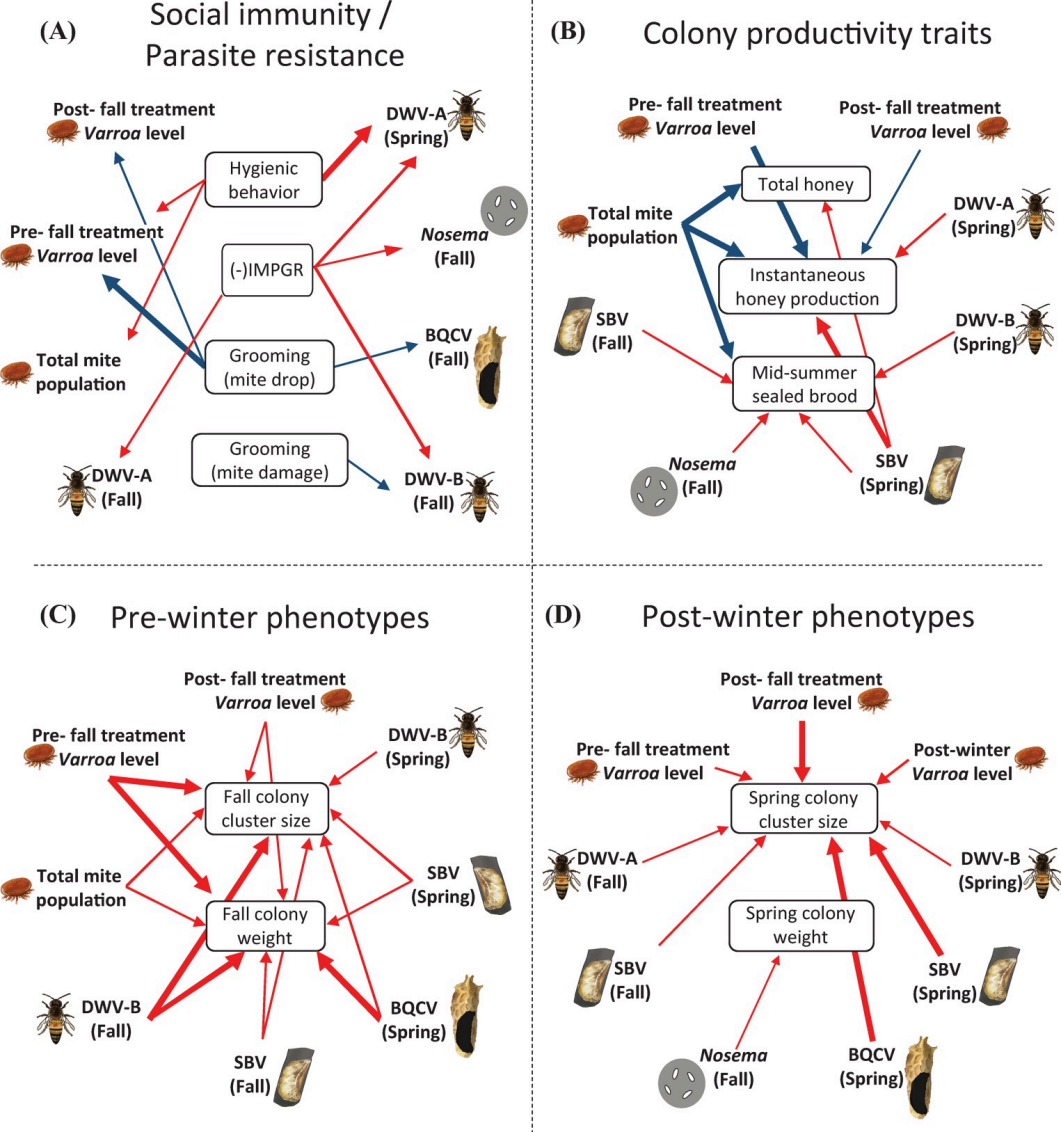
Graphical representation of linear regressions of colony phenotypes and pathogens. Arrows represent the effect of social immunity behaviors / parasite resistance (central boxes) on pathogen and parasite loads (A), the effect of pathogens and parasites on colony productivity traits (B), or the effects of pathogens and parasites on pre-winter (C) and post-winter (D) colony phenotypes. Positive relationships are illustrated with blue arrows and negative relationships with red arrows. Thicker arrows represent relationships that were observed in both experimental years, while thin arrows indicate that relationships observed only in one of the two experimental years. Only statistically significant relationships are shown. Refer to Table 4 for estimates of coefficients and statistical output.

The significance of regression models varied between years. In total, 49 models showed a significant association between the dependent and independent variable, with 17 showing a significant relationship in both years (Fig 4 and Table 4).

**Table 4:** Linear regression model summaries for the 2016–17 (year 1) and 2017–18 (year 2) datasets.

Year	Response~Explanatory Variable (fixed)	Random effects	Estimate	SEM	DF	*t*-value	*P*-value
**1**	**pre-fall treatment *Varroa* level ~ hygienic behavior**	tier + fall colony size	-0.01	0.01	692	-1.97	0.049
**1**	**total mite population ~ hygienic behavior**	-	-0.3	0.12	293	-2.48	0.014
**1**	**DWV-A spring ~ hygienic behavior**	tier + fall colony size	-1.41	0.6	558	-2.35	0.019
**2**		-	-2.26	0.97	241	-2.32	0.021
**2**	**pre-fall treatment *Varroa* level ~ IMPGR**	-	0.11	0.03	284	4.35	<0.0001
**1**	**total mite population *~* IMPGR**	-	2.02	0.22	295	9.06	<0.0001
**2**		-	3.9	0.22	293	17.79	<0.0001
**2**	**nosema *~* IMPGR**	-	8.92	2.3	283	3.88	0.0001
**2**	**DWV-A fall *~* IMPGR**	-	8.23	2.52	289	3.26	0.001
**2**	**DWV-B fall *~* IMPGR**	-	8.59	1.6	261	5.35	<0.0001
**2**	**DWV-A spring *~* IMPGR**	-	7.42	2.68	233	2.77	0.006
**1**	**pre-fall treatment *Varroa* level ~ grooming (mite drop)**	-	1.8	0.45	241	4	0.0001
**2**		-	0.87	0.25	246	3.44	0.001
**1**	**post-fall treatment *Varroa* level ~ grooming (mite drop)**	-	1.33	0.47	244	2.8	0.006
**1**	**total mite population ~ grooming (mite drop)**	-	-37.29	5.97	253	-6.25	<0.0001
**2**		-	-10.42	2.58	262	-4.04	0.0001
**1**	**BQCV fall ~ grooming (mite drop)**	-	83.24	22.9	250	3.63	0.0003
**1**	**DWV-B fall ~ grooming (mite damage)**	tier + fall colony size	1.06	0.53	460	1.98	0.048
**1**	**total honey ~ total mite population**	-	2.35	0.24	299	9.86	<0.0001
**2**		-	1.66	0.27	298	6.26	<0.0001
**2**	**total honey ~ SBV spring**	-	-0.24	0.06	226	-4.22	<0.0001
**1**	**instantaneous honey production ~ pre-fall treatment *Varroa* level**	tier + fall colony size	0.7	0.31	561	2.24	0.025
**2**		-	0.76	0.35	255	2.21	0.03
**1**	**instantaneous honey production ~ post-fall treatment *Varroa* level**	tier + fall colony size	0.3	0.15	541	2.02	0.044
**1**	**instantaneous honey production ~ total mite population**	-	0.11	0.02	201	6.01	<0.0001
**2**		-	0.2	0.03	239	6.35	<0.0001
**1**	**instantaneous honey production ~ DWV-A spring**	tier + fall colony size	-0.01	0.003	436	-2.45	0.015
**1**	**instantaneous honey production ~ SBV spring**	tier + fall colony size	-0.01	0.004	434	-2.57	0.01
**2**		-	-0.02	0.01	189	-2.94	0.004
**1**	**mid-summer sealed brood ~ total mite population**	-	0.32	0.06	244	5.77	<0.0001
**2**		-	0.15	0.06	288	2.59	0.01
**1**	**mid-summer sealed brood ~ nosema**	-	-0.02	0.01	320	-2.7	0.007
**1**	**mid-summer sealed brood ~ SBV fall**	-	-0.02	0.01	279	-2.05	0.04
**1**	**mid-summer sealed brood ~ DWV-B spring**	-	-0.02	0.001	173	-2.11	0.04
**1**	**mid-summer sealed brood ~ SBV spring**	-	-0.05	0.01	214	-3.96	0.0001
**1**	**fall cluster size ~ pre-fall treatment *Varroa* level**	tier	-4.88	1.07	446	-4.58	<0.0001
**2**		-	-2.83	0.68	294	-4.16	<0.0001
**1**	**fall cluster size ~ post-fall treatment *Varroa* level**	tier	-4.36	0.69	445	-6.3	<0.0001
**1**	**fall cluster size ~ total mite population**	-	0.24	0.08	297	3.03	0.003
**1**	**fall cluster size ~ DWV-B fall**	tier	-0.03	0.01	324	-2.33	0.02
**2**		-	-0.03	0.01	294	-2.64	0.009
**1**	**fall cluster size ~ SBV fall**	tier	-0.04	0.01	471	-3.56	0.0004
**1**	**fall cluster size ~ BQCV spring**	tier	-0.08	0.01	412	-5.48	<0.0001
**1**	**fall cluster size ~ DWV-B spring**	tier	-0.03	0.01	388	-2.16	0.03
**1**	**fall cluster size ~ SBV spring**	tier	-0.04	0.01	410	-2.72	0.007
**1**	**fall colony weight ~ pre-fall treatment *Varroa* level**	tier	-0.68	0.15	451	-4.45	<0.0001
**2**		-	-0.29	0.08	293	-3.78	0.0002
**1**	**fall colony weight ~ post-fall treatment *Varroa* level**	tier	-0.42	0.1	447	-4.2	<0.0001
**1**	**fall colony weight ~ total mite population**	-	0.03	0.01	293	3.08	0.002
**1**	**fall colony weight ~ DWV-B fall**	tier	-0.004	0.002	477	-2.39	0.02
**2**		-	-0.003	0.001	298	-2.46	0.015
**1**	**fall colony weight ~ SBV fall**	tier	-0.01	0.002	470	-5.24	<0.0001
**1**	**fall colony weight ~ BQCV spring**	tier	-0.005	0.002	407	-2.87	0.004
**2**		-	-0.005	0.001	241	-3.38	0.0009
**2**	**fall colony weight ~ SBV spring**	-	-0.004	0.001	183	-3.53	0.0005
**1**	**spring cluster size ~ DWV-A fall**	tier	-0.03	0.01	360	-2.79	0.006
**1**	**spring cluster size ~ SBV fall**	tier	-0.06	0.01	416	-4.2	<0.0001
**1**	**spring cluster size ~ BQCV spring**	tier	-0.09	0.02	406	-5.15	<0.0001
**2**		-	-0.09	0.02	235	-4.5	<0.0001
**1**	**spring cluster size ~ DWV-B spring**	tier	-0.05	0.01	398	-3.67	0.0003
**1**	**spring cluster size ~ SBV spring**	tier	-0.06	0.02	406	-3.49	0.0005
**2**		-	-0.04	0.02	181	-2.24	0.026
**2**	**spring cluster size ~ pre-fall treatment *Varroa* level**	-	-3.22	1.25	258	-2.57	0.011
**1**	**spring cluster size ~ post-fall treatment *Varroa* level**	tier	-3.58	1.25	258	-2.84	0.005
**2**		-	-14.27	4.74	157	-3.01	0.003
**1**	**spring cluster size ~ post-winter *Varroa* level**	tier	-5.42	1.44	397	-3.76	0.0002
**1**	**spring colony weight ~ nosema**	tier	-0.003	0.001	399	-2.51	0.01

Estimated coefficients of linear regressions were used to identify associations between colony‐level phenotypes and pathogen loads. The response and explanatory variables for each model, as well as random effects (tier = IM or SM), standard error of the mean (SEM), degrees of freedom (DF), t‐values and p‐values are also reported. Only significant results are listed.

#### Social immunity/parasite resistance

High hygienic behavior levels were associated with low final mite populations and high pre-fall treatment *V*. *destructor* levels in year 1 and low DWV-A levels in the spring of both years. Out of the six pathogens or parasites with which IMPGR was shown to have a significant positive association, two were measurements of *V*. *destructor* levels (pre-fall treatment *Varroa* levels and total mite population) and three were *V*. *destructor*-transmitted virus abundances (fall DWV-A, fall DWV-B, and spring DWV-A) (Fig 4; Table 4). High IMPGR (*i*.*e*., high mite population growth and thereby lower *Varroa* resistance) was further associated with higher *Nosema* spp. infections. In addition, we found grooming behavior to be negatively associated with total mite population; this was expected as total mite population data was used in the calculation of mite fall. Furthermore, high levels of grooming (mite fall) were associated with high levels of pre-fall treatment *Varroa* levels, post-fall treatment *Varroa* levels and fall levels of BQCV, while the proportion of mite damage was positively associated with DWV-B levels in the fall.

#### Productivity traits

Similar to our correlation results, we found that *V*. *destructor* variables were positively associated with honey production variables (Fig 4; Table 4). High pre- and post-fall treatment *Varroa* levels were associated with high instantaneous honey production. Total mite population was positively associated with both honey production traits (*i*.*e*., total honey and instantaneous honey production), as well as mid-summer sealed brood population. Conversely, high levels of the *V*. *destructor*-transmitted viruses DWV-A and B were associated with low instantaneous honey production and mid-summer sealed brood population, respectively. SBV had a negative association with all three productivity traits. A significant relationship was observed between fall SBV levels and mid-summer sealed brood population, and spring levels with all three productivity traits. Finally, increased fall *Nosema* spp. levels were associated with decreases in brood production in one of two years.

#### Pre- and post- winter phenotypes

Seven pathogens were negatively associated with colony cluster size in the fall and spring (*i*.*e*., pre- and post-fall treatment *Varroa* levels, DWV-B, SBV and BQCV spring levels and SBV fall level) (Fig 4; Table 4). Additionally, high fall levels of DWV-B and DWV-A were associated with low fall and spring colony cluster sizes, respectively. Fall colony weight data showed significant associations with the same pathogens found to have a significant relationship with fall cluster size, with the only exception being spring levels of DWV-B. Spring colony weight only showed a significant relationship with fall *Nosema* spp. levels, suggesting that the former may not be an informative variable of colony health status.

## Discussion

The increased mortality of honey bee colonies in recent years has significantly impacted the beekeeping industry in Canada, and worldwide. Canadian beekeepers report that high pathogen/parasite infestation levels are one of the top causes of elevated colony losses [4]. In this large-scale study, where over 1500 colonies of diverse genetic background were assessed over two years, we investigated inter-correlations between individual pathogens, parasites and colony-level phenotypes, as well as across these factors. We also compared regional differences across these datasets and compared virus abundance levels before and after wintering. Our main objectives were to better understand the relationships among disease factors, colony level phenotypes and associated productivity traits, as well as to elucidate the influence of social immunity behaviors on colony pathogen load. Our results show that colonies expressing high levels of three out of four of the social immunity behaviors studied (hygienic behavior, *Varroa* resistance behavior and grooming-related mite damage) were associated with low levels of pathogens/parasites, including viruses, *Nosema* spp., and mites. We have also shown that high viral and *Nosema* spp. loads are associated with low colony productivity traits. Finally, our data further illustrate that five of six pathogens/parasites we studied showed a strong negative relationship with pre-winter colony phenotypes and were negatively associated with the outcomes of colonies the following spring. The information generated regarding the incidence and abundance of pathogens, colony phenotypes and their inter-correlations will enable beekeepers and queen producers to make informed decisions when managing and selecting colonies to be healthy, productive and well-adapted to the Canadian climate.

Social insects have evolved remarkable behaviors at the societal level (social immunity) to counter several pathogen/parasite challenges, which can reduce colony-level disease, and improve colony health. One of the most extensively studied honey bee social immunity behaviors is hygienic behavior [20,45,46]. Hygienic bees play a crucial role in the population dynamics of pathogens and pests in the hive as bees carrying this trait are able to detect and remove diseased and/or parasitized brood. Our results support previous research and confirm the negative association between high levels of hygienic behavior and *V*. *destructor* population levels [20,24,26,30,47,48]. Additionally, our findings reveal a negative relationship between hygienic behavior and DWV-A levels. The parasitic *Varroa* mite is an effective vector of several honey bee viruses, including viruses of the DWV/VDV-1 clade [9,49,50]. Therefore, infestation levels of mite-transmitted viruses, such as DWV-A in hygienic colonies, may be affected as a result of the removal of mite-parasitized brood from the colony. The significant relationship between hygienic behavior and spring levels of DWV-A in both experimental years, and the less consistent association between hygienic behavior and mite population, could suggest that hygienic bees preferentially target removal of brood parasitized by DWV-infected mites, or pupae infected with DWV, as opposed to simply removing mite-infested brood [25,26].

In addition to hygienic behavior, bees have other defense mechanisms against *Varroa* mites. This ectoparasitic mite, particularly in combination with viral infection, is currently one of the most significant threats to the beekeeping industry. Despite its serious impact on honey bee health, several studies worldwide have documented honey bee populations that are able to manage and/or mitigate mite population growth without beekeeping intervention. Following a previously-published protocol [34], we assessed the Instantaneous Mite Population Growth Rate (IMPGR) as a measurement of a colony’s natural mite resistance. High IMPGR (*i*.*e*., low *Varroa* resistance) was positively associated with fall levels of DWV-A, DWV–B, *Nosema* spp., as well as spring levels of DWV-A during the second year of this experiment. These results are consistent with the strong positive association between DWV titers and *Varroa* mite infection levels [49,51,52], which is in great part due to the virus’ ability to replicate within its mite host and be horizontally transmitted to other members of the colony by *Varroa*. The association between *Nosema* spp. and *Varroa* mites is, however, less clear. Previous studies have also found a positive relationship between these two parasites [6,53–57], but others failed to observe any significant relationship [58]. Studies suggest that peritrophic membrane permeability increases following *Varroa* infestation, which increases bee susceptibility to *Nosema* infection [54]. Additionally, *Nosema* infection may increase susceptibility to *Varroa* infestation due to its effect on fat body stores and bee behavior [54]. Nonetheless, these results support the benefit of using IMPGR in genetic selection programs to improve the population’s natural resistance to mites as well as lower viral titers and, possibly, *Nosema* spp. infection levels.

The removal and damage of parasitic mites by a worker bee from its own body (auto-grooming), or from a nest-mate’s body (allo-grooming), are major behavioral mite-resistance traits in the Asian honey bee, *Apis cerana* [27], the original host of *Varroa destructor* [28]. These mite-resistance behaviors are also documented in the mite’s western honey bee host, *Apis mellifera*, particularly as a long-term tolerance mechanism to *Varroa* in African honey bee subspecies [29]. Grooming is an important defensive mechanism against mites, as evidenced by the relationship between damaged mites and colony infestation level for both African and European honey bees [21,59–64]. Our results did indicate a significant positive relationship between natural mite fall and phoretic *Varroa* infestation levels, consistent with previous investigations [65,66]. Despite this, our data failed to show any significant relationship between damaged mites and the mite infestation indices measured (*i*.*e*., pre- and post-fall treatment *Varroa* levels and post-winter *Varroa* levels), suggesting that our first and second cohort genetic stock was primarily composed of a non mite-grooming population or that mite damage is not necessary for successful grooming to occur. Data from our first year also indicated a positive association of natural mite fall with fall BQCV levels, as well as an association between autumn levels of DWV-B and the proportion of damaged mites. It is possible that these relationships are a result of the mite association with both BQCV and DWV as a mechanical vector, and with DWV as a biological vector. Further research is needed using high mite-grooming European honey bee colonies to elucidate the relationship between mite damage and mite population levels, or more specifically, mite-transmitted viral levels. Based on our findings, the reliability of grooming behavior measurements for the selection of Varroa “resistant” colonies would likely be improved when paired with another varroa resistance parameter.

Honey bees are a host for several viruses. Here, we quantified the abundance of four viruses that have been associated with colony collapse and overwinter mortality: DWV-A, DWV-B, BQCV and SBV. Most honey bee viruses do not cause an overt infection, which precludes field diagnoses and allows viral infections to go unnoticed by beekeepers. Although several studies have linked high viral infections to reductions in colony survival and bee lifespan [18,67–73], their effects on colony strength, productivity and pre- and post- winter performance remains largely unknown. Here, we show that high SBV levels found in colonies in the autumn, as well as SBV and DWV-B levels found in the spring, are strongly associated with reduced brood population prior to winter. Although SBV can be found in adult bees, young larvae (*i*.*e*., 2-day-old) are the most susceptible stage to SBV infection. SBV infection is lethal to infected larvae, resulting in death due to the failure of larvae to pupate [5], which negatively impacts the colony’s brood population. Similarly, DWV infection also has the potential to negatively impact brood populations. Deformed Wing Virus can be detected in both brood and adult bees, in colonies with or without overt DWV symptoms such as shrivelled wings and decreased body size. Desai and Currie [73] did not find a correlation between SBV and fall or spring populations but spring levels were a significant predictor of colony death which is in agreement with our results. In infected brood, DWV infection has been shown to cause death in the pupal stage [49], which can in turn affect colony population size. Our results also show a negative relationship of SBV and DWV-A on colony productivity traits (*i*.*e*., honey production). Iqbal and Mueller [13] demonstrated that artificial infection of adult bees with DWV via a vectorial-like transmission causes an impairment of sensory responsiveness and associated olfactory learning. They concluded that clinical symptoms of DWV infection, therefore, include a negative impact on individual bee performance and, consequently, colony performance and productivity. In addition, early SBV studies have shown that SBV infection in young adults affects individual behavior. Infection with Sacbrood virus decreases the appetite of adult bees for pollen, which may cause a series of downstream behavioral changes (*i*.e., abandoning of hive duties) and shortened lifespan [15,18,68,74].

High levels of *Nosema* spp. were found to be associated with low mid-summer sealed brood populations. *Nosema* spp. spores, when ingested by adult bees through contaminated food, water or when cleaning infected cells, infect and replicate within midgut cells [75]. *Nosema* spp. infections can cause precocious foraging and lead to decreased lifespan [14]. *Nosema* spp. infections of adult bees disrupt the social dynamics of the hive and subsequently impact colony growth and brood production (*i*.*e*., decreased nurse bee population and infection of the queen), as reported in several studies [7,16,17].

The observed positive association between mid-summer sealed brood cells and mite infestation level was not surprising as this has also been observed by others [76–79]. *Varroa* mites have a non-reproductive phase on adult bees for movement between hosts and/or feeding [80], and a reproductive phase inside drone or worker brood cells [81]. When brood is not scarce, there is a positive relationship between brood cells and mite infestation in the brood [76–79]. We also found that the mid-summer sealed brood population has a positive correlation with honey production, as observed during both experimental years, and in previous studies [76,82,83]. Therefore, the positive association between honey production (total and instantaneous) and *Varroa* mite infestation data is most likely due to the positive influence of sealed brood on the adult bee population [76].

Several studies document the negative effect of high levels of viruses and *Varroa* mites on honey bee winter survival [10,12,58,67,73,84,85]. As important as managing pathogen loads are, beekeeping practices that ensure appropriately-sized colonies in the fall also promote greater wintering success [84,86,87]. Despite our current knowledge that high pathogen levels and small colony sizes (in regards to population and food stores) are predictive markers of winter colony loss, no large scale studies have thoroughly investigated the relationship between pathogens and colony cluster size and strength across multiple climatic regions. We collected two colony metrics (*i*.*e*., colony weight and cluster size) prior to- and after winter, as important colony phenotypes associated with winter survival. During both experimental years, we observed a significant negative correlation between several pathogens and our pre- and post- winter colony measurements. It is uncertain whether high pathogen loads are the cause of low colony size and strength, or if colonies that are already weak provide a favourable environment for pathogens to actively multiply. Dainat et al. [84] showed that colonies with lower adult populations in the fall had significantly higher levels of DWV and *Varroa*, compared with colonies with higher adult populations. Moreover, workers from colonies that did not survive the winter had reduced lifespan, and significantly higher levels of DWV and *Varroa* mites in the late fall, suggesting an effect of high levels of DWV on winter bee life span, ultimately resulting in colony death or a small colony size in the spring [67]. Our results show negative associations of not only DWV and *Varroa*, but also SBV and BQCV levels, with fall colony weight and cluster size. In the spring, only one pathogen was (negatively) associated with colony weight (*Nosema* spp.), while eight pathogens/parasites were (negatively) associated with colony cluster size. This lack of relationship between spring colony weight and pathogens is likely due to weak populations in the spring having a considerable amount of food stores remaining contributing to higher colony weights. This colony phenotype makes the data indistinguishable between strong colonies and weak colonies, because of the large amount of food stored in the latter. Therefore, using spring cluster size is a more appropriate tool for selection of colonies in the early spring than using spring colony weight.

Our regression models show that some fall phenotypes can be significantly associated with spring viral levels, but not by the level of the same virus in the fall (*i*.*e*., hygienic behavior and spring DWV-A; instantaneous honey production and spring DWV-A; mid-summer sealed brood and spring DWV-B; fall colony cluster size or colony weight and spring BQCV). We hypothesize that this is due to DWV levels in unhealthy colonies not decreasing during the winter at a similar rate as observed in healthy colonies, and remaining significantly higher in the spring [67]. As for BQCV levels, our data suggest that BQCV may not always decrease appreciably during the winter, or may even potentially increase from fall to spring. Similarly, Desai and Currie found that DWV titre decreases significantly over winter in outdoor wintered colonies but increases slightly in colonies wintered outdoors whereas BQCV tends to increase overwinter in both wintering environments. We suggest that this epidemiological pattern of DWV and BQCV in unhealthy colonies establishes a more distinguishable relationship during the spring than the fall levels of each of these two viruses, and may be more useful for selection purposes.

The differences in model significance between years suggest that unknown biotic and abiotic factors may play a role in pathogen population growth and the relationship between each pathogen population and colony phenotypes/traits. Suitable environmental conditions may not occur every year to the same extent, thereby having a significant impact on pathogen replication and/or colony phenotype/trait expression [39]. The relative abundance of viruses may also change among years. For example, in our two year study we found little prevalence of IAPV and no impact on colony health, yet earlier studies in Canada indicated it was very common with prevalence ranging from 13 to 70% occurrence in healthy colonies and it was correlated with spring population size in indoor wintering environments [73,88]. The reasons for the apparent reduction in the presence of this economically important virus are not known.

In conclusion, variation in the ability of a colony to manage and/or mitigate mite population growth and other pathogens can be explained by the queen source genetics, the environment, and the interaction of these factors on the expression of social immunity traits [e.g. hygienic behavior, grooming behavior and IMPGR; 89]. Our results suggest that hygienic behavior may have a strong influence on the expression of viral levels such as DWV-A. In contrast to other studies showing the effect of grooming behavior on mite population growth, our findings revealed no significant negative associations between grooming (mite drop or mite damage) and *Varroa* infestation levels, although a significant negative correlation was observed between mite drop and total mite population on year 1, and mite damage and both post-fall treatment and post-winter *Varroa* level on year 2. Colonies with low mite population growth in our study were associated with low *Nosema* spp. and viral levels (*i*.*e*., DWV-A and DWV-B). Our data also expands our current knowledge of the effects of pathogens on colony productivity, size and strength, showing a significant negative association between several pathogens and economically important colony phenotypes. It is particularly important to emphasize the implication that high viral levels may have on colony traits. *Varroa* mites, when associated with viruses, can pose a dangerous threat to the colony, as infestations may be able to kill a colony at a lower threshold than in the absence of viruses [90]. Although it is simple for a beekeeper to measure mite loads in the field, assessment of viral levels can be very costly and consequently it is not a common practice in beekeeping operations. As an alternative, our results provide the tools for beekeepers to select for traits/phenotypes that have been demonstrated to have an association with low viral levels, such as high hygienic behavior and low *Varroa* mite population growth, as well as managing larger colonies in preparation for winter.

## Supporting information

S1 Table Locus (common) names, primer names, sequences and references for genes tested via real-time PCR. Key: DWV-A, deformed wing virus A; DWV-B, deformed wing virus B; BQCV, black queen cell virus; IAPV, Israeli acute paralysis virus; SBV, sacbrood virus; RP49, ribosomal protein 49; RPS5, ribosomal protein S5; bp, base pair.
(DOCX)
