# Supplementary material for: Phenomic analysis of the honey bee pathogen-web and its dynamics on colony productivity, health and social immunity behaviors
Source: PLoS One. 2022 Jan 31;17(1):e0263273. doi: 10.1371/journal.pone.0263273 (PMC8803170; doi:10.1371/journal.pone.0263273)
Supplement: S1 Table — Key: DWV-A, deformed wing virus A; DWV-B, deformed wing virus B; BQCV, black queen cell virus; IAPV, Israeli acute paralysis virus; SBV, sacbrood virus; RP49, ribosomal protein 49; RPS5, ribosomal protein S5; bp, base pair. (DOCX) [file pone.0263273.s001.docx]

| Target | Primer | Sequence (5’-3’) | Amplicon size (bp) | Reference |
| --- | --- | --- | --- | --- |
| *Lotmaria passim* | LpCytb_F2 | AGTATGAGCAGTAGGTTTTATTATA | 146 | [1] |
|  | LpCytb_R | GCCAAACACCAATAACTGGTACT |  |  |
| DWV-A | DWV-F8668 | TTCATTAAAGCCACCTGGAACATC | 136 | [2] |
|  | DWV-B8757 | TTTCCTCATTAACTGTGTCGTTGA |  |  |
| DWV-B (VDV) | VDV-F2 | TATCTTCATTAAAACCGCCAGGCT | 140 | [3] |
|  | VDV-R2a | CTTCCTCATTAACTGAGTTGTTGTC |  |  |
| BQCV | BQCV-qF7893 | AGTGGCGGAGATGTATGC | 294 | [2] |
|  | BQCV-qB8150 | GGAGGTGAAGTGGCTATATC |  |  |
| IAPV | IAPV-F1aF | GCGGAGAATATAAGGCTCAG | 587 | [4] |
|  | IAPV-F1a R | CTTGCAAGATAAGAAAGGGGG |  |  |
| SBV | SBV-qF3164 | TTGGAACTACGCATTCTCTG | 335 | [2] |
|  | SBV-qB3461 | GCTCTAACCTCGCATCAAC |  |  |
| RP49 | RP49-qF | AAGTTCATTCGTCACCAGAG | 205 | [2] |
|  | RP49-qB | CTTCCAGTTCCTTGACATTATG |  |  |
| RPS5 | RPS5-F | AATTATTTGGTCGCTGGAATTG | 105 | [4] |
|  | RPS5-R | TAACGTCCAGCAGAATGTGGTA |  |  |

**References**

1. Schwarz RS, Bauchan GR, Murphy CA, Ravoet J, De Graaf DC, Evans JD. Characterization of two species of trypanosomatidae from the Honey Bee *Apis mellifera*: *Crithidia mellificae* Langridge and McGhee, 1967 and *Lotmaria passim* n. gen., n. sp. *J Eukaryot Microbiol*. 2015;62(5):567-583. doi:10.1111/jeu.12209.

2. Locke B, Forsgren E, Fries I, de Miranda JR. Acaricide treatment affects viral dynamics in *Varroa destructor*-infested honey bee colonies via both host physiology and mite control. *Appl Environ Microbiol*. 2012;78(1):227-235. doi:10.1128/AEM.06094-11.

3. Mcmahon DP, Fürst MA, Caspar J, Theodorou P, Brown MJF, Paxton RJ. A sting in the spit: Widespread cross-infection of multiple RNA viruses across wild and managed bees. *J Anim Ecol*. 2015;84(3):615-624. doi:10.1111/1365-2656.12345.

4. vanEngelsdorp D, Evans JD, Saegerman C, et al. Colony collapse disorder: A descriptive study. *PLoS One*. 2009;4(8). doi:10.1371/journal.pone.0006481.
